# Clinical characteristics of pregnant women with COVID-19 in Japan: a nationwide questionnaire survey

**DOI:** 10.1186/s12884-021-04113-9

**Published:** 2021-09-18

**Authors:** Tatsuya Arakaki, Junichi Hasegawa, Akihiko Sekizawa, Tomoaki Ikeda, Isamu Ishiwata, Katsuyuki Kinoshita, the Japan Association of Obstetricians and Gynecologists (JAOG), Tomoaki Ikeda, Koyo Yoshida, Isamu Ishiwata, Akihiko Sekizawa, Yasushi Kuribayashi, Shunji Suzuki, Kazuhiko Ochiai, Hirokatsu Kitai, Kentaro Shimura, Kaoru Kimura, Ken Ishitani, Junichi Hasegawa, Masaji Nagaishi, Tatsuya Arakaki, Gen Ishikawa, Susumu Oka, Osamu Samura, Hiroaki Tanaka, Hiroshi Ishikawa, Takao Kobayashi, Yasumasa Ohno, Kayo Osada, Yukiko Kawana, Hiroyuki Seki, Masahiko Nakata, Koji Hashii, Satoshi Hayashi, Takeshi Murakoshi, Katsuyuki Kinoshita

**Affiliations:** 1 Department of Obstetrics and Gynecology, Showa University School of Medicine, 1-5-8 Hatanodai, Shinagawa-ku, Tokyo, 142-8666 Japan; 2 Department of Obstetrics and Gynecology, St. Marianna University School of Medicine, 2-16-1 Sugao, Miyamae-ku, Kawasaki, Kanagawa 216-8511 Japan; 3 Department of Obstetrics and Gynecology, Mie University School of Medicine, 2-174 Edobashi, Tsu, Mie 514-8507 Japan; 4 Ishiwata Obstetrics and Gynecology Hospital, 1-4-21 Kamimito, Mito, Ibaraki 310-0041 Japan; 5 Seijo Kinoshita Hospital, 6-13-20 Seijo, Setagaya-ku, Tokyo, 157-0066 Japan

**Keywords:** COVID-19, Pregnancy, Delivery

## Abstract

**Background:**

Few reports have presented an overall view of pregnant women with coronavirus disease 2019 (COVID-19) across an entire country and throughout the entire gestation period. Furthermore, no such reports are available for Japan. We examined the clinical characteristics and outcomes of pregnant women with COVID‑19 on a national scale in Japan.

**Methods:**

A nationwide questionnaire-based survey for all 2,185 maternity services in Japan was conducted between July and August 2020. Information regarding maternal characteristics and epidemiological, clinical, treatment, and perinatal outcomes of pregnant women diagnosed with COVID-19 between 16 January and 30 June 2020 were collected. Main outcome measures were incidence of pregnant women with COVID-19 and infant infection, positive rate of the universal screening test for asymptomatic pregnant women, identification of infection route and rates of maternal death, and severe cases.

**Results:**

Responses from 1,418 institutions were assessed (65% of all delivery institutions in Japan). Seventy-two pregnant women were reported to have been diagnosed with COVID-19. The positive rate of the universal screening test for severe acute respiratory syndrome coronavirus 2 (SARS-CoV-2) among asymptomatic pregnant women was 0.03% (2/7428). The most common route of infection was familial (57%). Fifty-eight pregnant women with COVID-19 were symptomatic, of whom five (8.6%) had a severe infection and one died (a tourist). Severe respiratory symptoms, oxygen administration, and pneumonia were frequently reported in the third trimester and postpartum period compared with in early pregnancy (22.2% vs 2.5% [*P* = 0.03], 38.9% vs 7.5% [*P* = 0.01], and 50.0% vs 7.5% [*P* < 0.001], respectively). All pregnant women with COVID-19 underwent caesarean sections, regardless of symptoms. There were no SARS-CoV-2 transmissions to newborns.

**Conclusions:**

In Japan, the number of cases of COVID-19 infection in pregnant women is very low. Compared with early pregnancy, late pregnancy may be a risk factor for exacerbation of symptoms and familial transmission is the most common route of infection. The importance of infection prevention should be emphasised, especially in women in late pregnancy, their families, and any cohabitants.

## Background

The World Health Organisation (WHO) declared the coronavirus disease (COVID‑19) outbreak as a pandemic in March 2020 [1]. In Japan, the first case of COVID-19 was reported on 16 January 2020 [2]. In response to the spread of infection, the Japanese government began conducting polymerase chain reaction (PCR) testing for severe acute respiratory syndrome coronavirus 2 (SARS-CoV-2) on all patients with fever and respiratory symptoms or those who had been in close contact with infected people. On 7 April 2020, a state of emergency was declared, which lasted until 25 May 2020, in which schools were closed, business activities were suspended, and external movements for non-essential reasons were prohibited. As a result, the first wave of infections in Japan, which began in early April 2020, was over by the end of June 2020 [3].

For respiratory viral infections such as influenza, pregnant women are known to be at a high risk for severe illness with the highest risk occurring in later pregnancy [4, 5]. However, it is not known if SARS-CoV-2 infections show a similar trend. Although the number of cases of COVID-19 is increasing worldwide, the clinical features of COVID-19 in pregnant women remain unknown. While reports to date have been presented as case series, there are few studies that have examined pregnant women throughout pregnancy on a national scale [6]. Therefore, an overview of pregnant women with COVID-19 across an entire country and throughout an entire gestation period, has not been presented. We investigated the characteristics and outcomes of pregnant women with COVID-19 in Japan and utilised the findings to propose future prevention and treatment strategies.

## Methods

The Japan Association of Obstetricians and Gynaecologists (JAOG) conducted a nationwide questionnaire-based survey examining the effects of COVID-19 on maternity services between July and August 2020. A questionnaire with a cover letter outlining the purpose of the study was mailed to the Director or the Chief obstetrician in foetal-maternal medicine of all 2,185 delivery institutions in Japan. Survey Responses to the questionnaire were reported by the physician in charge at each institution, either through an online platform (google form) or by fax, using anonymised information obtained from medical records. The missing important information about COVID-19 patients was reconfirmed to each medical institution by e-mail or fax. The questionnaire consisted of two parts: a standard questionnaire and a detailed questionnaire. The standard questionnaire included questions on the number of pregnant and postpartum women with COVID-19 who were being managed within each unit, as well as whether universal testing of asymptomatic pregnant women had been performed between 16 January and 30 June 2020. In instances where a pregnant woman with COVID-19 was reported, the detailed questionnaire investigated the maternal characteristics, course of onset, symptoms, epidemiological history, clinical course, and maternal and perinatal outcomes of each case. All the patient data were anonymised.

A confirmed case of COVID-19 was defined as having a positive real-time reverse transcriptase (RT)-PCR SARS-CoV-2 assay from nasal and pharyngeal swab specimens or having respiratory compromise in the presence of characteristic radiographic changes of COVID-19. Pregnant women with a history of symptoms or potential exposure to COVID-19 were tested for SARS-CoV-2. In some hospitals, universal screening of all pregnant women was performed. Nasal and pharyngeal swabs were obtained from almost all newborns born to infected mothers, and the samples were subsequently tested using RT-PCR. Computed tomography (CT) of pregnant women was performed at the discretion of the doctor, with pneumonia being diagnosed based on CT findings.

Pregnant women with COVID-19 were categorised as symptomatic or asymptomatic based on the presence or absence of symptoms prior to admission and during hospitalisation. The severity of disease was defined as severe or critical. Severe cases were defined as cases with severe respiratory symptoms (having one of the following: respiratory rate > 30/min; percutaneous oxygen saturation < 93%; or ratio of arterial oxygen partial pressure to inspired oxygen fraction < 300) [7]. Critical cases were defined as those requiring admission to the intensive care unit (ICU) or having respiratory failure and requiring mechanical ventilation.

### Statistical analyses

Data were analysed using IBM SPSS Statistics for Mac, version 25.0 (IBM Corp., Armonk, NY, USA). Nonparametric continuous variables were compared using the Mann–Whitney *U* test. Categorical variables were compared using the Chi-squared test and Fisher’s exact test (two-sided). Statistical significance was defined as *P*-value < 0.05.

## Results

Questionnaires were sent to 2,185 medical facilities with maternity services. Responses were received from 1,418 (64.9%) facilities with a recorded total of 611,444 deliveries in 2019 (71.1% of all deliveries in Japan). The universal SARS-CoV-2 screening for asymptomatic pregnant women was conducted at 158 (11%) facilities, with a total of 7,428 people tested, of which two were positive, for a positivity rate of 0.02%.

**Fig. 1:**
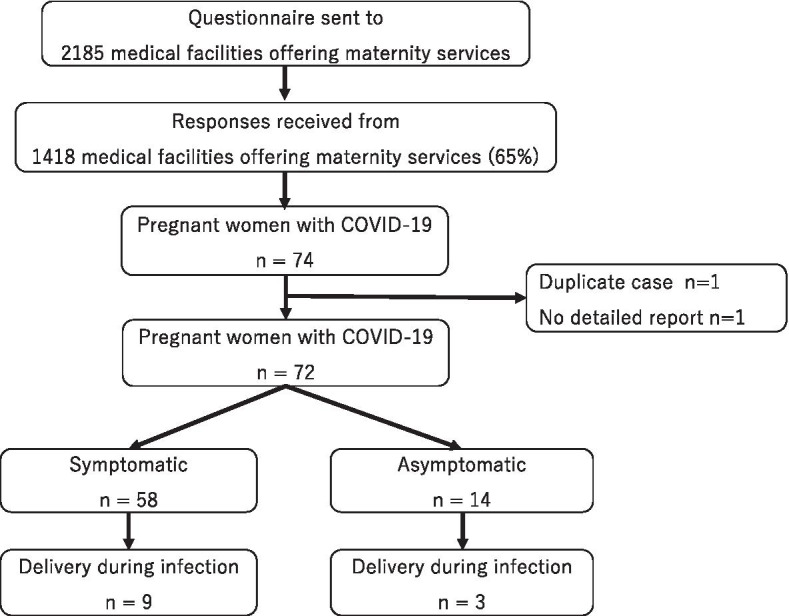
Study flow diagram

The study flow is shown in Fig. 1. Seventy-four pregnant women with confirmed COVID-19 were treated in 48 institutions between 16 January and 30 June 2020. Of these, one case was a duplicate record and another had missing data. Hence, 72 women with complete data were included in this analysis, of whom 58 (80.6%) were symptomatic and 14 (19.4%) were asymptomatic.

The maternal characteristics of symptomatic and asymptomatic COVID-19 patients are shown in Table 1.

**Table 1:** Maternal characteristics of symptomatic and asymptomatic COVID-19 patients

	**Symptomatic patients (*n* = 58)**	**Asymptomatic patients (*n* = 14)**	***P*-value**
***Maternal age***			
Median (interquartile range)	31 (27–36)	32 (25–33)	0.41
Under 20	0 (0%)	0 (0%)	> 0.99
20–29	20 (34.5%)	6 (42.9%)	0.56
30–39	33 (56.9%)	8 (57.1%)	0.99
over 40	5 (8.6%)	0 (0%)	0.58
***Approach for diagnosis***			
Symptom	57 (98.3%)	0 (0%)	< 0.001
Contact with confirmed or suspected patients	1 (1.7%)	12 (85.7%)	< 0.001
Universal screening	0 (0%)	2 (14.3%)	0.04
***Pregnancy period at diagnosis***			
1st trimester	12 (20.7%)	3 (21.4%)	> 0.99
2nd trimester	28 (48.3%)	4 (28.6%)	0.18
3rd trimester	15 (25.9%)	5 (35.7%)	0.51
Postpartum	3 (5.2%)	2 (14.3%)	0.25
***Epidemiological history***			
Familial infection	33 (56.9%)	8 (57.1%)	0.99
Community-acquired	5 (8.6%)	3 (21.4%)	0.18
Visiting and staying in infected areas	4 (6.9%)	0 (0%)	0.58
Nosocomial infection	2 (3.4%)	1 (7.1%)	0.48
Workplace infection	1 (1.7%)	2 (14.3%)	0.10
Unknown	13 (22.4%)	0 (0%)	0.06
***Comorbidities***			
Gestational diabetes	5 (8.6%)	0 (0%)	0.58
Preeclampsia	1 (1.7%)	0 (0%)	> 0.99
Asthma	3 (5.2%)	0 (0%)	> 0.99
***Course of pregnancy***^a^			
Pregnancy ongoing	29 (50.0%)	7 (50.0%)	> 0.99
Delivery	21 (36.2%)	5 (35.7%)	0.97
Delivery during infection	9 (15.5%)	3 (21.4%)	0.69
Spontaneous abortion	2 (3.4%)	0 (0%)	> 0.99
Induced abortion	1 (1.7%)	0 (0%)	> 0.99
***Prognosis of mother***			
Recovered without subsequent complication	57 (98.3%)	14 (100%)	> 0.99
Death	1 (1.7%)	0 (0%)	> 0.99

Data indicate n (%)

^a^ Excluding 1 maternal death, 1 case of missing data, and 5 cases of puerperium diagnosis

The median maternal ages of symptomatic and asymptomatic patients were 31 (interquartile range [IQR], 27–36) and 32 (IQR, 25–33) years, respectively. Among asymptomatic patients, 85.7% had been tested after having been in contact with confirmed or suspected individuals and 14.3% were tested during universal screening. The majority of symptomatic patients were diagnosed during the second trimester (48.3%), while the majority of asymptomatic patients were diagnosed during the third trimester (35.7%). In both groups, the most common route of infection was familial (56.9% for symptomatic patients and 57.1% for asymptomatic patients). Only one asymptomatic patient (1.7%), a tourist from Europe, died due to COVID-19.

The maternal clinical presentation of symptomatic COVID-19 patients is shown in Table 2.

**Table 2:** Maternal clinical presentation of symptomatic COVID-19 patients (*n* = 58)

***Signs and symptoms***	
Fever	41 (70.7%)
37.0–38.0 (°C) ^a^	26 (47.2%)
38.0–39.0 (°C) ^a^	13 (23.6%)
Cough, respiratory distress, or sore throat	39 (67.2%)
Taste and smell disorders	23 (39.7%)
Fatigue	17 (29.3%)
Muscular pain	4 (6.9%)
Headache	4 (6.9%)
Nasal discharge and nasal congestion	2 (3.4%)
Diarrhea	2 (3.4%)
Itchy throat	1 (1.7%)
Severe respiratory symptoms	5 (8.6%)
***Chest CT examination***	
Performed during pregnancy	17 (29.3%)
Radiologically confirmed pneumonia	12 (20.7%)
***Treatments***	
Hospitalization	48 (82.8%)
Time from symptom onset to hospitalization (days) ^b^	6 (2–10)
Time from hospitalization to worsening of symptoms (days) ^c^	0 (0–4)
Oxygen administration	10 (17.2%)
Intensive care unit admission	3 (5.2%)
Invasive mechanical ventilation	1 (1.7%)

Data indicate n (%) or median (interquartile range); CT: computed tomography

^a^Excluding 3 cases with missing data

^b^Excluding 3 cases with missing data

^c^Excluding 9 cases with missing data

For 58 symptomatic COVID-19 patients, the most common symptoms were fever (70.7%) and cough, respiratory distress, or sore throat (67.2%). Five patients (8.6%) had severe respiratory symptoms. Seventeen women (29.3%) underwent a chest CT scan during pregnancy, and 12 (20.7%) had radiologically confirmed pneumonia. Forty-eight (82.8%) were hospitalised and the time from symptom onset to hospitalisation was a median of 6 days (IQR, 2–10). Ten women (17.2%) required oxygen administration and three (5.2%) were admitted into the ICU. One woman (1.7%) needed mechanical ventilation and later died.

A total of 24 infected women delivered infants during the study period. Of these, 12 deliveries occurred during active infection while the others occurred after recovery.

Perinatal outcomes of COVID-19 patients who delivered during infection and after recovery are shown in Table 3.

**Table 3:** Perinatal outcomes of COVID-19 patients who delivered during active infection and after recovery

	**During active infection (*n* = 12)**				**After recovery (*n* = 14)**	***P*-value**^b^
	**Total**	**Symptomatic patients (*n* = 9)**	**Asymptomatic patients (*n* = 3)**	***P*-value**^a^		
***Timing of delivery***						
Gestational age at delivery (weeks and days)	38w2d (37w1d-38w5d)	38w4d (36w5d-38w6d)	37w5d (37w2d-39w4d)	0.93	38w5d (37w5d-39w2d)	0.43
Preterm birth	2 (16.7%)	2 (22.2%)	0 (0%)	> 0.99	1 (7.1%)	0.58
Due to iatrogenic causes	2 (16.7%)	2 (22.2%)	0 (0%)	> 0.99	0 (0%)	0.20
***Mode of delivery***^d^						
Spontaneous vaginal delivery	0 (0%)	0 (0%)	0 (0%)	> 0.99	5 (45.5%)	0.01
Induction of labour	0 (0%)	0 (0%)	0 (0%)	> 0.99	3 (27.3%)	0.09
Caesarean section	12 (100%)	9 (100%)	3 (100%)	> 0.99	3 (27.3%)	< 0.001
Due to obstetrical indications	1 (8.3%)	1 (11.1%)	0 (0%)		3 (27.3%)	
Due to concern about COVID-19	11 (91.7%)	8 (88.9%)	3 (100%)		0 (0%)	
***Neonatal outcome***^e^						
Apgar score < 7 at 1 min	0 (0%)	0 (0%)	0 (0%)	> 0.99	0 (0%)	> 0.99
Apgar score < 7 at 5 min	0 (0%)	0 (0%)	0 (0%)	> 0.99	0 (0%)	> 0.99
Birth weight (g)	3015 (2601–3109)	2950 (2549–3105)	3110 (2805–3109)	0.21	2907 (2377–3250)	0.53
Performed SARS-CoV-2 test	11 (100%)	9 (100%)	2 (100%)^c^	> 0.99	0 (0%)	< 0.001
Confirmed Positive	0 (0%)	0 (0%)	0 (0%)	> 0.99	-	-
Breastfeeding	1 (9.1%)	1 (11.1%)	0 (0%)^c^	> 0.99	10 (100%)	< 0.001
Mother-Neonate Separation	12 (100%)	9 (100%)	3 (100%)	> 0.99	0 (0%)	< 0.001

Data presented as n (%) or medians (interquartile ranges)

*SARS-CoV-2* severe acute respiratory syndrome coronavirus 2, *COVID-19* coronavirus disease 2019

^a^Symptomatic and asymptomatic patients who delivered during active infection were compared

^b^Patients during active infection and after recovery were compared

^c^Data was not available for one patient as delivery had just occurred at the time of data collection

^d^Excluding 3 cases with missing data of patients after recovery

^e^Excluding 4 cases (3 cases for birthweight) with missing data of patients after recovery

Nine women were symptomatic and three were asymptomatic. All 12 women underwent caesarean sections, with 11 of them performed owing to concerns about the effects of COVID-19 on pregnancy. All 12 women delivered live infants, and no severe neonatal asphyxia was observed. Eleven newborns were tested for SARS-CoV-2 using nasal and pharyngeal swabs, and all tested negative. None of the infants born to these women suckled; one infant received expressed breast milk, while the others were given formula milk. In all the cases, the mother and baby were separated while in hospital. On the other hand, pregnant women who delivered after recovering from infection were under normal obstetric care. There was no difference in gestational age at delivery compared to pregnant women during the active infection, but vaginal delivery and breastfeeding were more common, and there was no mother-neonate separation.

The maternal outcomes of symptomatic patients in each pregnancy period are shown in Table 4. Severe respiratory symptoms, oxygen administration, and radiologically confirmed pneumonia were frequently reported in the third trimester and postpartum period compared with those in early pregnancy (22.2% vs 2.5% [*P* = 0.03], 38.9% vs 7.5% [*P* = 0.01], and 50.0% vs 7.5% [*P* < 0.001], respectively).

**Table 4:** Maternal outcomes of symptomatic patients during each pregnancy period

	**1st or 2nd trimester (*n* = 40)**	**3rd trimester or puerperium (*n* = 18)**	***P*-value**
Respiratory rate > 30/min	1 (2.5%)	3 (16.7%)	0.08
SpO_2_ < 93%	1 (2.5%)	3 (16.7%)	0.08
PaO_2_/FiO_2_ ratio less than 300	1 (2.5%)	0 (0%)	> 0.99
Severe respiratory symptoms	1 (2.5%)	4 (22.2%)	0.03
Oxygen administration	3 (7.5%)	7 (38.9%)	0.01
Radiologically confirmed pneumonia	3 (7.5%)	9 (50.0%)	< 0.001

Data presented as n (%)

*SpO2* Percutaneous oxygen saturation, *PaO2* Arterial oxygen partial pressure, *FiO2* Inspired oxygen fraction

## Discussion

In this nationwide survey conducted in Japan, 72 COVID-19 cases were reported to have been diagnosed between 16 January and 30 June 2020 among pregnant women and the positive rate of the universal SARS-CoV-2 screening test among asymptomatic pregnant women was very low (0.03%). The most common route of infection was familial (57%). Severe respiratory symptoms, oxygen administration, and pneumonia were more commonly observed during late pregnancy than during early pregnancy, suggesting that COVID-19 may potentially be more severe during late pregnancy. All pregnant women infected with COVID-19 underwent caesarean section and no SARS-CoV-2 transmission was detected in the newborn. None of the infants born to these women suckled, regardless of the mothers’ symptoms.

There were approximately 305,722 deliveries in the aforementioned 6-month period in the institutions that participated in this survey. Therefore, the estimated incidence rate of COVID-19 over the study period was 23.6 per 100,000 pregnant women. The prevalence of COVID-19 among asymptomatic pregnant women during the study period was 0.03% (2/7428), which is much lower than the prevalence reported in the United States (1.5% and 13.7%)[8, 9]. The prevalence in asymptomatic pregnant women was as low as the estimated incidence rate, suggesting that there was no strong benefit to the screening tests. With the noted prevalence, expansion of the PCR testing for symptomatic pregnant women should be a priority. We believe that this approach may also be useful in preventing nosocomial infections. Among symptomatic COVID-19 patients, the rates of 8.6% severe respiratory symptoms, 5.2% ICU admission, and 1.7% need for ventilation and death were comparable to other reports [10, 11].

According to a study conducted in the United Kingdom, most pregnant women admitted to the hospital with COVID-19 symptoms are in the third trimester or peripartum period [6]. Additionally, a report from Spain found that the proportion of women with symptoms and those requiring hospitalisation was higher among women in the third trimester [12]. Maternal physiological adaptation of cardiovascular and respiratory systems during pregnancy and the associated immunological changes may result in reduced tolerance to respiratory infections and pneumonia during pregnancy, especially in the later stages [13, 14]. Our results, along with these reports, suggest that pregnant women with COVID-19 may experience more severe symptoms in the third trimester and during postpartum. Therefore, both pregnant women and healthcare providers need to be more cautious about COVID-19 during late pregnancy than during early pregnancy. In the present study, the time from symptom onset to hospitalisation was six days, with peak symptoms occurring on the day of admission. Shortening the duration from onset of symptoms to hospitalisation may allow interventions, especially in late pregnancy. There is still no consensus on the optimal treatment or selection of the best imaging modality for pregnant COVID-19 patients [15, 16]. Chest CT is recommended for use in patients with moderate to severe features of COVID-19 and patients with middle features but at risk for exacerbations [17]. Even for pregnant women, it is stated that chest CT should be performed when indicated and should not be delayed for fetal considerations [18]. Regarding treatment, it has been reported that for non-pregnant women, initiating medication in the early stages of the disease may reduce the severity and mortality [15, 19]. Depending on the severity of COVID-19, individualised approaches should be preferred, and management decisions should be made. Especially in pregnant women with COVID-19 whose respiratory status worsens during late pregnancy, it would be advisable to evaluate the disease status by chest CT and consider the use of drugs such as antivirals.

Nearly 60% of the cases were diagnosed or suspected to have a familial infection. It is possible that the emergency declaration by the Japanese government that led to a reduction in outdoor person-to-person contact, may have resulted in more pronounced familial infections. According to public data on the status of infection in Tokyo, which has the highest number of infected people in Japan, the proportion of pregnant women among all infected people from April to August 2020 was 0.53%. The proportion of pregnant women in the population of Tokyo in 2020 is estimated to be 1.89%. The proportion of pregnant women among all infected people is lower by more than one-third the proportion of pregnant women in the population. Therefore, it is presumed that pregnant women are taking appropriate measures to prevent infection. Under these circumstances, to prevent familial infection as a further infection control measure, not only pregnant women but also their families and any cohabitants need to be vigilant in ensuring infection prevention. To prevent familial clusters, isolation should be considered if someone in the family has symptoms or has been in contact with confirmed or suspected COVID-19 patients.

Although there was no SARS-CoV-2 transmission to the new-borns, all pregnant women with COVID-19 were delivered via caesarean sections, regardless of their symptoms. It has been suggested that the presence of COVID-19 alone is not an indication for a caesarean section and that the mode of delivery should follow obstetric indications [20]. Ferrazzi et al. proposed that vaginal delivery is appropriate in mild COVID-19 cases and that caesarean sections should be planned in severe cases [21]. In addition, a caesarean delivery has been reported to be associated with an increased risk of clinical deterioration [22]. Owing to few facilities with negative pressure delivery rooms in Japan, the Japan Society of Obstetrics and Gynecology (JSOG) has stated that caesarean sections may be selected for the purpose of shortening labour time, simultaneously securing medical resources, and preventing mother-to-child transmission [23]. This statement may have greatly influenced the choice of a caesarean section during the study period, which was in the early stages of COVID-19 in pregnant women in Japan. Currently, an increasing number of facilities have changed policies and are now opting for vaginal deliveries. The mode of delivery should be individually determined depending on maternal conditions.

Breast milk, which was expressed, was administered in only one asymptomatic case. The JSOG recommends the use of artificial milk because the breast milk could possibly contain the virus [23]. In contrast, it has been suggested that SARS-CoV-2 is unlikely to infect newborns via breast milk and the WHO recommends that mothers with suspected or confirmed COVID-19 should be encouraged to initiate or continue breastfeeding [24]. It has recently been reported that perinatal transmission is unlikely to occur, even during active maternal infection, with appropriate hygiene precautions. Therefore, when combined with effective parental education on infant infection control, it is recommended that mothers and newborns be placed in the same room and breastfed directly [15, 25].

A major strength of this study is that it was the first rapid nationwide questionnaire survey of pregnant women with COVID-19 in Japan. Therefore, the results are extremely valuable for understanding the current situation of infection and determining future measures in Japan. A limitation is that the analysis was based on a questionnaire survey that asked for experiences and reported the clinical courses of COVID-19 from medical records. Therefore, there could be potential recall bias in the outcomes, especially maternal outcomes, and missing information. We did not collect detailed data, such as racial background, blood test results, treatment, or changes in symptoms before and after delivery. Furthermore, the number of pregnant women with COVID-19 is relatively small. Therefore, further accumulation of detailed data is needed to examine the impact of infection and its severity on perinatal outcomes. The response rate for this rapid survey was 65%; however, the questionnaire survey may not fully reflect the true situation of the survey population compared with data obtained from the medical report system directly, so the representativeness of the research may be limited, and the total number of infected patients could have been underestimated because the status of all facilities is not available. It is necessary to build a national surveillance system for prospective observation.

## Conclusion

In Japan, the number of cases of COVID-19 in pregnant women is very low. Our result does not indicate that pregnant women are more likely to become severely ill, but it does indicate that late pregnancy may be a risk factor for the exacerbation of the symptoms compared with early pregnancy and that familial transmission is the most common route of infection. The importance of infection prevention should be emphasised, especially in women in late pregnancy, their families, and any cohabitants.

## Data Availability

The datasets used and/or analysed during the current study are available with the corresponding author on reasonable request.

## Abbreviations

COVID-19: Coronavirus disease 2019
SARS-CoV-2: Severe acute respiratory syndrome coronavirus 2
WHO: World Health Organisation
PCR: Polymerase chain reaction
JAOG: Japan Association of Obstetricians and Gynaecologists
RT: Reverse transcriptase
CT: Computed tomography
ICU: Intensive care unit
IQR: Interquartile range
JSOG: Japan Society of Obstetrics and Gynecology
